# Serious adverse drug events associated with psychotropic treatment of bipolar or schizoaffective disorder: a 17-year follow-up on the LiSIE retrospective cohort study

**DOI:** 10.3389/fpsyt.2024.1358461

**Published:** 2024-04-03

**Authors:** Petra Truedson, Michael Ott, Lisa Wahlström, Robert Lundqvist, Martin Maripuu, Krister Lindmark, Ingrid Lieber, Ursula Werneke

**Affiliations:** ^1^ Department of Clinical Sciences, Psychiatry, Sunderby Research Unit, Umeå University, Umeå, Sweden; ^2^ Department of Public Health and Clinical Medicine, Medicine, Umeå University, Umeå, Sweden; ^3^ Department of Psychiatry, Sunderby Hospital, Luleå, Sweden; ^4^ Department of Public Health and Clinical Medicine, Sunderby Research Unit, Umeå University, Luleå, Sweden; ^5^ Department of Clinical Sciences, Psychiatry, Umeå University, Umeå, Sweden; ^6^ Department of Clinical Sciences, Danderyd University Hospital, Karolinska Institutet, Stockholm, Sweden

**Keywords:** adverse drug events, psychotropic drugs, lithium, incidence, intoxication, neuroleptic malignant syndrome, bipolar disorder, serotonin syndrome

## Abstract

**Introduction:**

Mood stabilisers and other psychotropic drugs can lead to serious adverse drug events (ADEs). However, the incidence remains unknown. We aimed to (a) determine the incidence of serious ADEs in patients with bipolar or schizoaffective disorders, (b) explore the role of lithium exposure, and (c) describe the aetiology.

**Methods:**

This study is part of the LiSIE (Lithium—Study into Effects and Side Effects) retrospective cohort study. Between 2001 and 2017, patients in the Swedish region of Norrbotten, with a diagnosis of bipolar or schizoaffective disorder, were screened for serious ADEs to psychotropic drugs, having resulted in critical, post-anaesthesia, or intensive care. We determined the incidence rate of serious ADEs/1,000 person-years (PY).

**Results:**

In 1,521 patients, we identified 41 serious ADEs, yielding an incidence rate of 1.9 events per 1,000 PY. The incidence rate ratio (IRR) between ADEs with lithium present and causally implicated and ADEs without lithium exposure was significant at 2.59 (95% CI 1.20–5.51; p = 0.0094). The IRR of ADEs in patients <65 and ≥65 years was significant at 3.36 (95% CI 1.63–6.63; p = 0.0007). The most common ADEs were chronic lithium intoxication, oversedation, and cardiac/blood pressure-related events.

**Discussion:**

Serious ADEs related to treatment of bipolar (BD) or schizoaffective disorder (SZD) were uncommon but not rare. Older individuals were particularly at risk. The risk was higher in individuals exposed to lithium. Serum lithium concentration should always be checked when patients present with new or unclear somatic symptoms. However, severe ADEs also occurred with other mood stabilisers and other psychotropic drugs.

## 1 Introduction and aims

In many cases, bipolar (BD) and schizoaffective disorder (SZD) require long-term mood-stabilising treatment to prevent relapse into affective episodes or suicide. If the clinical course is complex, such as in rapid cycling disorder, several mood stabilisers may be combined. During acute events, additional anti-psychotics and other sedatives are often needed. Some patients receive anticholinergics to counter extrapyramidal adverse effects of antipsychotics. Other patients receive antidepressants or, when needed, *pro re nata* (PRN) medications. Finally, individuals with BD or SZD also have an increased risk of other psychiatric and somatic comorbidities (1). These may require additional pharmacological treatment, which is why many individuals with BD or SZD will receive combination rather than monotherapy during their lifetime. Up to one-third of outpatients with BD may receive treatment with ≥3 psychotropic drugs. Being white, age >50 years, subtherapeutic dosing, lower treatment adherence, more extensive comorbidity, and a greater history of suicide attempts have been identified as factors that increase the risk of polypharmacy (2).

Mood stabilisers and other psychotropic drugs, despite their therapeutic utility, can give rise to adverse drug events (ADEs). Serious ADEs can result in emergency medical conditions. The risk of such serious ADEs may be increased by a combination of drugs that interact, a high-dose administration of psychotropic drugs, or use of parenteral formulations. But serious ADEs may even occur idiosyncratically, i.e., non-dose dependently (3–5). Drugs with narrow therapeutic index, such as lithium, can give rise to intoxications (6). At present, the incidence of such serious ADEs remains unknown. Neither is it known how lithium compares to other mood stabilisers in terms of ADEs. On one hand, lithium may directly cause serious ADEs through intoxications. On the other hand, lithium may reduce the need for other psychotropic drugs, which, on their own or in combination, could lead to serious ADEs. Prospective studies that assess serious ADEs in sufficient detail are difficult to conduct. Information from register studies is insufficient because register studies do not have enough information to distinguish association from causality. Finally, studies that only explore serious ADEs retrospectively or explore psychiatric comorbidity in the context of acute or intensive care admission may overestimate the incidence.

The aims of this study were for patients with BD or SZD to (a) determine the incidence of serious ADEs, (b) compare the incidence rates of serious ADEs caused by lithium with those of ADEs caused by other psychotropic medications, and (c) describe the aetiology of the serious ADEs identified.

## 2 Materials and methods

### 2.1 Study design

This study was part of LiSIE (Lithium—Study into Effects and Side Effects), a retrospective cohort study based on longitudinal medical records review from Northern Sweden (7–9). LiSIE was set up to identify the best long-term treatment options for patients with bipolar disorder (BD) or related condition by exploring the effects and potential adverse effects of lithium compared to those of other mood stabilisers.

### 2.2 Ethics and consent procedures

All participants were informed about the nature of the study in writing and provided verbal informed consent. The consent was documented in our research files, dated, and signed by the research worker who obtained the consent. In accordance with the ethics approval granted, deceased patients were also included. The consent procedures were concluded at the end of 2012. The cohort was locked at this point; no new patients were included in the study thereafter. LiSIE adheres to the Declaration of Helsinki guidelines and has been approved by the Regional Ethics Review Board at Umeå University, Sweden (DNR 2010-227-31M, DNR 2011-228-32M, DNR 2014-10-32M, DNR 2018-76-32M). The study is conducted according to the STrengthening the Reporting of OBservational studies in Epidemiology (STROBE) checklist (**Appendix**). The LiSIE study has been described in several previous studies (7, 8) and is summarised below.

### 2.3 Sample

LiSIE invited all adults in the regions of Västerbotten and Norrbotten who had either received a diagnosis of BD (ICD10 F31) or schizoaffective disorder (SZD) (ICD10 F25) according to the 10th revision of the International Statistical Classification of Diseases and Related Health Problems (10) or who had used lithium as a mood stabiliser between 1997 and 2011.

### 2.4 Patient selection and inclusion criteria

The current study included patients from the Norrbotten region having received a diagnosis of either BD or SZD on at least two occasions at least 180 days apart. In line with the ICD-10 classification, we also included patients under the BD category, when they had been diagnosed with at least one manic and one depressive event. For this study, we screened all events of critical, post-anaesthesia, or intensive care documented in the medical records. We only included events related to an unintended serious ADE involving a psychotropic drug in its own right or in terms of a drug interaction.

### 2.5 Exclusion criteria

For the whole LiSIE study, we excluded patients in whom, after manual medical record validation, a diagnosis of schizophrenia or personality disorder was more likely than BD or SZD. For the current study, we excluded patients in whom ADE occurred during care under forensic services; we had not applied for ethical approval to access these records.

### 2.6 Outcome

The primary outcome was the number of serious ADEs. We defined events as serious ADEs when they (a) concerned an adverse effect or drug interaction involving at least one psychotropic drug (**Table 1**), (b) led to a critical, post-anaesthesia, or intensive care, (c) were not a result of an intentional overdose, and (d) could not be better explained by another aetiology unlinked to the psychopharmacologic treatment. We then calculated the incidence in events per 1,000 person years (PY) overall and stratified by lithium exposure and age (<65 and ≥65 years).

**Table 1:** Psychotropic drugs reviewed in the study.

Substance/substance class	ATC code
Antiepileptics	N03
Anti-Parkinson’s drugs – Anticholinergic agents	N04A
Psycholeptics	N05
Psychoanaleptics	N06
Other nervous system drugs – Drugs used in addictive disorders	N07B

ATC, Anatomical Therapeutic Chemical code.

### 2.7 Exposure parameters

The main exposure parameter was psychotropic drugs. To characterise the serious ADEs, we checked for use of PRN, polypharmacy, and use of parenteral psychotropic drugs, the latter either in the form of long-acting injectable antipsychotics (LAI) or acute injections. Polypharmacy was defined as concurrent use of ≥3 psychotropic medicines (2). For inpatients, we were not able to establish doses because these fluctuated, and documentation was not complete. For outpatients, we recorded doses given on the prescription at the time of the ADE. We also explored the use of somatic drugs that could have interacted and somatic comorbidities that could have been related to the ADE. Other exposure parameters included age, sex, and type of underlying mood disorder. For subcategories of mood disorder, we relied on previous validation of the LiSIE cohort that had explored how diagnoses would have looked according to DSM-5, BD (296.4, 296.80, 296.89) or SZD (295.7) (11). This validation had used medical records until 31 December 2015. We also checked for serum lithium concentrations and serum creatinine concentrations at the time of the serious ADE. We also checked serum potassium concentrations as a risk factor for arrythmias (12). Finally, we divided patients in four groups according to the role lithium exposure might have played: (A) lithium exposure at the time and causally implicated, (B) lithium exposure at the time but not causally implicated, (C) no lithium exposure at the time, and (D) no lithium exposure at the time but previous lithium causally implicated.

### 2.8 Time in the study

The outcome of serious ADEs was determined over a 17-year period from 1 January 2001 to 31 December 2017. In accordance with the set-up of the study, we did not consider treatment times before the age of 18 years. Observation time stopped if information in the case records suggested that the patient had moved out of the catchment area or had died before 31 December 2017. In our observation time-frame, for each patient, we then counted PY separately for time periods with and without lithium treatment, and time periods for age <65 or ≥65 years.

### 2.9 Chart review and validation

For the outcomes and exposure variables, we retrospectively reviewed the medical records of all eligible patients from 1 January 2001 to 31 December 2017. From the medical records, we manually validated serious ADEs and the concurrent lithium treatment. At that point, based on the information in the medical records, we determined the most likely cause of the ADE. In patients treated with lithium at the time, we evaluated whether lithium was (a) causally implicated in the ADE, (b) used at the time but not implicated in the ADE, or (c) not used. For all ADEs, we also recorded somatic comorbidities present at the time, which could have possibly contributed to the event. Two authors with psychiatric background (PT and UW) conducted the validation of ADEs. When uncertain, they consulted with a third author with a medical background (MO). To ensure that we did not miss any potential drug interactions, including cytochrome 450 (CYP)-mediated interactions, we used the “Janusmed” interaction checker (13). Janusmed interaction is an online drug/drug interaction (DDI) database published by the health authority of Stockholm Region and used by health authorities and medical professionals all across Sweden (14, 15). The interactions are classified into four categories from A to D with ascending severity. Category A signifies an interaction with no clinical relevance; B, an interaction with unclear or varying clinical relevance; C, a clinically relevant interaction that can, for instance, be handled with dose adjustment; and D, a clinically relevant interaction that should be avoided. For DDI possibly related to an ADE, we only considered C and/or D interactions, i.e., interactions rated as clinically relevant (13).

### 2.10 Control for bias and missing data

We had controlled for selection bias in the whole LiSIE study with key parameters available in anonymised form. These included age and sex. Where applicable, we also controlled for maximum recorded concentrations of lithium and creatinine. In accordance with the ethics approval granted, we had compared these parameters for consenting and non-consenting patients. No significant differences were found between the two groups (16, 17). The data were complete for included patients for the defined outcome. For some patients, not all medications could be derived from the prescription module. For these, we could not accurately establish the dose. For some drugs, we could not establish with certainty involvement in the severe ADE. For instance, for PRN medications, like non-steroidal-anti-inflammatory drugs (NSAID), it was not always clear from the case records whether the patient had actually taken the drug or not. Such we reported under “other potentially contributing medications” in the table listing the ADEs.

### 2.11 Statistical analysis

The data were anonymised before analysis. Then, the data were analysed descriptively. We calculated the incidences for serious ADEs with and without lithium being causally implicated and for age <65 or ≥65 years. We then calculated the incidence rate ratio (IRR) and 95% confidence intervals (CI). We also described serious ADEs regarding relevant exposure parameters stratified by lithium exposure. As the sample size was small, we used non-parametric methods. We used Fisher’s exact test to compare sex and treatment setting and Mann–Whitney U test to compare age. We did not conduct any further multivariate analysis to avoid overfitting, i.e., fitting too many variables in relation to the small sample size. The data were processed with SPSS version 27.0 (IBM, Armonk, NY, USA) and MedCalc Software Ltd. (Version 20.116). The significance level was set at a p-value of 0.05 throughout.

## 3 Results

### 3.1 Baseline characteristics

A total of 1,521 patients were included. Of these, 945 (62.1%) were female and 576 (37.9%) were male; 1,298 (85.3%) had a diagnosis of BD and 223 (14.7%) a diagnosis of schizoaffective disorder. During the whole review period, 841 (55.3%) patients had been exposed to lithium at any time. In terms of observation time, there were 21,977 PY available, 5,586 PY with lithium exposure, 16,391 PY without lithium exposure, 19,038 PY for age <65 years, and 2,939 PY for age ≥65 years.

**Figure 1:**
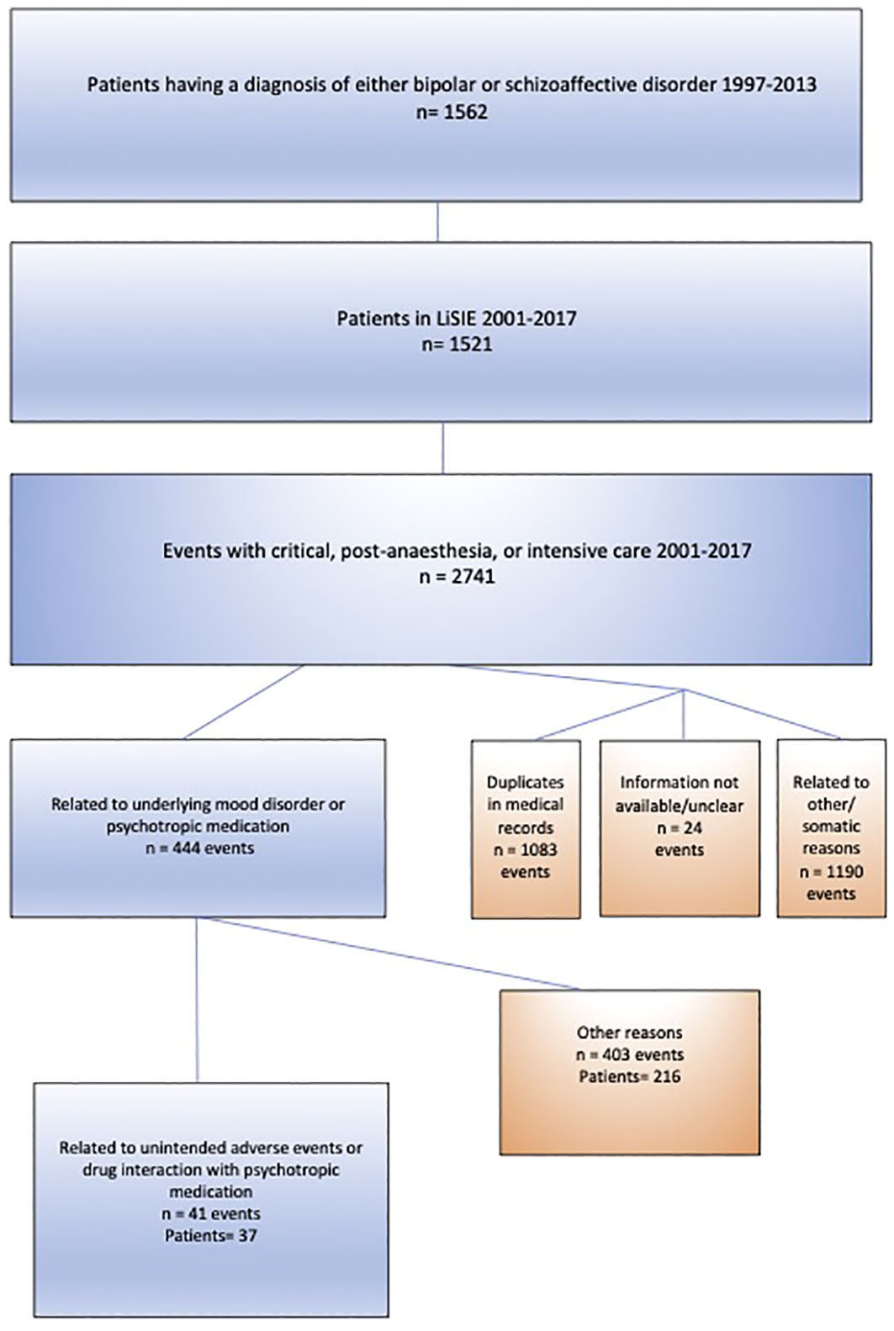
Selection of study sample.

In total, there were 37 patients, 14 men and 23 women, who had experienced 41 events of serious ADE (**Figure 1**). Thirty-four patients had one event, two patients had two events, and one had three events. Of all serious ADEs, 34.1% concerned patients ≥65 years, 56.1% had lithium treatment at the time, and 51.2% had second-generation antipsychotics. Polypharmacy was present in 70.7%, PRN medication in 58.5%, LAIs in 12.2%, and 12.2% had received an acute parenteral injection at the time (**Table 2**).

**Table 2:** Summary characteristics of the 41 serious adverse drug events.

	N (%)
Age, years	
18–40	8 (19.5)
41–64	19 (46.3)
>65	14 (34.1)
Sex	
Male	14 (34.1)
Female	27 (65.9)
Main diagnosis	
Bipolar disorder (BD)	31 (75.6)
Schizoaffective disorder (SZD)	10 (24.4)
Type of psychiatric treatment at the time	
Outpatient	28 (68.3)
Inpatient	13 (31.7)
Lithium at the time	
Concurrent lithium treatment	23 (56.1)
Concurrent treatment with antiepileptics^a,b^	
Valproate	6 (14.6)
Lamotrigine	3 (7,3)
Carbamazepine	2 (4,9)
Concurrent treatment with antipsychotics^a,b^	
Second-generation antipsychotics (SGA)	21 (51.2)
First-generation antipsychotics (FGA)	16 (39.0)
Long-acting injectable (LAI)	5 (12.2)
Other treatment charcteristics	
Polypharmacy: ≥3 concurrent psychotropic drugs^b^	29 (70.7)
PRN medications^b^	24 (58.5)
Acute intramuscular injection^a,c^	5 (12.2)

PRN, pro re nata, when needed.

a Events might have involved more than one type of drug at the same time.

b In one event, complete information regarding prescribed medications was not available in the medical records.

c One injection given 10 days before the event was excluded.

### 3.2 Incidence of adverse drug events

The overall ADE incidence was 1.9/1,000 PY. Although 23 events had lithium treatment at the time, lithium treatment was causally implicated in 16 cases. In one event, the ADE was due to lithium-induced nephrogenic diabetes insipidus caused by previous lithium treatment (**Table 3**).

**Table 3:** Incidence of ADE caused by lithium.

	All	Group A	Group B	Group C	Group D^a^
Lithium treatment at time of ADE		Yes	Yes	No	No
Lithium causally implicated		Yes	No	No	Yes
N	*41*	15	8	17	1
PY	*21,977*	5,586	5,586	16,391	N/A
Incidence	*1.9/1,000*	2.7/1,00	1.4/1,000	1.0/1,000	N/A

ADE, adverse drug event; N, number; PY, person years; N/A, not applicable.

a Diabetes insipidus related to previous lithium treatment. Not on lithium at time of ADE.

The IRR between groups A and C was significant at 2.59 (95% CI 1.20–5.51; p = 0.0094). The IRR between groups B and C was non-significant at 1.38 (95% CI 0.52–3.38; p = 0.4517). The incidence of serious ADE in patients <65 years was 1.4/1,000 PY. The incidence of serious ADE in patients ≥65 years was 4.8/1,000 PY. The IRR between the age groups was significant at 3.36 (95% CI 1.63–6.63; p = 0.0007).

#### 3.2.1 Subgroup analysis

Of the 16 events with ADE related to lithium, 81.3% occurred in women, and 25.0% in inpatients. The median age was 64.5 (range 43–80) years. Of the 25 events with ADE not related to lithium, 56.0% occurred in women and 36.0% in inpatients. The median age was 54.0 (range 24–87) years. There were no statistically significant differences in sex (p = 0.176) or inpatient status at the time of the ADEs (p = 0.513). There was a significant difference regarding age distribution (p = 0.024).

### 3.3 Adverse drug event characteristics

Chronic lithium intoxication was the commonest cause of a serious ADE at 36.6%, followed by oversedation at 24.3%, and cardiac and blood pressure-related causes at 21.9%. The remaining seven events included two cases of neuroleptic malignant syndrome (NMS), one of which was confirmed, whereas the other was suspected; two cases of acute dystonia (acute dystonic reactions) with a risk of airway obstruction; one case of suspected paradoxical seizure; one case of hypernatraemia due to lithium-induced nephrogenic diabetes insipidus; and one case of serotonin syndrome. In all of the 15 events of chronic lithium intoxication, elevated creatinine levels were present. General weakness, drowsiness, and confusion were the commonest symptoms. Eight of the 10 events of oversedation occurred in the context of drug combinations from at least two substance classes, such as benzodiazepines or related substances, opioids, or antipsychotics. Two of the nine events with cardiac and blood pressure-related causes were due to QT prolongations. In one of these events, methadone precipitated torsades de pointes (TdP). In the other event of QT prolongation, sertraline was the only drug implicated. There was one case of malignant hypertension in the context of venlafaxine treatment. Four, or possibly five, events were related to possible hypotension, two of which resulted in a collapse. The two cases of NMS each involved a combination of two different antipsychotics, where at least one was injectable. The two cases of acute dystonia also concerned antipsychotics. In the first case, the acute dystonic reaction was precipitated when LAI zuclopenthixol was added to olanzapine. The other case of acute dystonic reaction was caused by haloperidol given orally. The case of serotonin syndrome concerned a combination of sertraline and tramadol. Of the 41 ADEs, 20 events had at least one category C or D DDI, which was possibly implicated. Of these, 19 ADEs had C interactions, and one had a D interaction. In six events, there were more than one C interactions present. Eleven C-interactions concerned combinations of lithium with either diuretics, NSAIDs, or angiotensin-converting enzyme (ACE) inhibitors. In one of the lithium-related ADEs, the DDI was identified as the sole cause. In the other 10 events, the DDI could have been a contributory cause. In 14 of the 16 lithium-related ADEs, a relevant somatic comorbidity was also present. For two ADEs, a CYP interaction was implicated and classified as a C interaction. Both were related to CYP2D6 inhibition of zuclopenthixol by levomepromazine (**Table 4**).

**Table 4:** Type of reaction and involved medications in 41 events of serious adverse drug events documented between 2001 and 2017.

Event	Age group	Psychiatric treatment status	Implicated medications^a^	Other potentially contributing medications	Clinically relevant DDI present according to Janusmed^b^	Recorded somatic comorbidity at the time of event	Serum K (mmol/L) (range)	Serum creatinine (μmol/L) (range)	Serum Li (mmol/L)^c^	Serious ADE	
										Type	Symptoms
1	41–64	Outpatient	Lithium Naproxen	Enalapril Furosemide	C: Lithium–naproxen C: Lithium–furosemide C: Lithium–enalapril	Bowel pains and nausea days before, for which patient self-initiated treated with naproxen	6.0 (3.6–4.6)	671 (50–90)	1.58	Chronic Li-intox	Kidney failure, anorexia, bowel pains
2	41–64	Outpatient	Lithium	Furosemide	C: Lithium–furosemide	COPD, obesity, pneumonia, heart failure	4.1 (3.6–4.6)	168 (50–90)	2.50	Chronic Li-intox	Unconsciousness, respiratory depression
3	41–64	Inpatient	Lithium			Suspected dehydration due to hot weather	3.9 (3.6–4.6)	107 (60–105)	2.56	Chronic Li-intox	Confusion, disorientation, ataxia
4	41–64	Inpatient	Lithium	Furosemide Naproxen	C: Lithium–furosemide C: Lithium–naproxen		2.7 (3.6–4.6)	98 (45–90)	3.25	Chronic Li-intox	Confusion, ataxia, anorexia, drowsiness
5	41–64	Outpatient	Lithium	Bendroflumethiazide	C: Lithium–bendroflumethiazide	Unclear seizures in the past	3.3 (3.6–4.6)	115 (45–90)	2.56	Chronic Li-intox	Seizure, agitation, anxiety
6	41–64	Outpatient	Lithium	Bendroflumethiazide Furosemide	C: Lithium–bendroflumethiazide C: Lithium–furosemide	Infection week before	2.8 (3.6–4.6)	106 (45–90)	2.78	Chronic Li-intox	Drowsiness, leg instability and general weakness, blurred speech
7	41–64	Outpatient	Lithium		None	Metastatic intestinal cancer, recent chemotherapy	3.8 (3.6–4.6)	218 (45–90)	2.68	Chronic Li-intox	Drowsy, loss of balance, polyuria
8	41–64	Outpatient	Lithium	Bendroflumethiazide	C: Lithium–bendroflumethiazide	Suspected dementia, general weakness since years back	3.0 (3.6–4.6)	161 (60–100)	1.93	Chronic Li-intox	Worsen general weakness, anorexia, suspected diarrhoea
9	≥65	Outpatient	Lithium		None	Reduced oral intake weeks before	3.7 (3.6–4.6)	112 (50–90)	2.62	Chronic Li-intox	Confusion, leg instability, general weakness, diarrhoea
10	≥65	Outpatient	Lithium	Enalapril Furosemide Diclofenac	C: Lithium–diclofenac C: Lithium–enalapril C: Lithium–furosemide	Heart failure, ischaemic heart disease	4.7 (3.6–4.6)	122 (60–105)	1.42	Chronic Li-intox	Leg instability and general weakness
11	≥65	Inpatient	Lithium		None	Severe depression, anhedonia, dehydration	4.2 (3.6–4.6)	100 (45–90)	1.72	Chronic Li-intox	Fear of arrhythmias, need of rehydration
12	≥65	Outpatient	Lithium	Enalapril Furosemide Metoprolol	C: Lithium–enalapril C: Lithium–furosemide	Post CABG, atrial fibrillation	5.4 (3.6–4.6)	260 (60–105)	1.47	Chronic Li-intox	Bradycardia
13	>65	Outpatient	Lithium	Enalapril	C: Lithium–enalapril	Recent fracture, pain, fever	5.6 (3.6–4.6)	166 (50–90)	1.96	Chronic Li-intox	Confusion, “worsened psychosis”
14	≥65	Outpatient	Lithium	Furosemide	C: Lithium–furosemide		3,7 (3.6–4.6)	167 (45–90)	3,13	Chronic Li-intox	Collapse, instability leading to fall, rhabdomyolysis
15	≥65	Outpatient	Lithium	Bendroflumethiazide	C: Lithium–bendroflumethiazide	Previous episode of bradycardia, infection/pneumonia	4,6 (3.6–4.6)	670 (50–90)	1,81	Chronic Li-intox	Loss of consciousness, respiratory depression, dehydration, fever
16	≥65	Inpatient	Lithium		None	Lithium-induced nephrogenic diabetes insipidus, primary hyperparathyroidism, severe depression, anhedonia, anorexia	3.8 (3.6–4.6)	225 (50–90)	No treatment at time	Hypernatremia due to lithium induced NDI	Hypernatraemia, lethargy
17	41–64	Outpatient	Alprazolam	Diazepam Disulfiram Zolpidem Morphine	C: Disulfiram–diazepam	COPD, hepatitis C, chronic pain	4.3 (3.6–4.6)	N/A	N/A	Oversedation	Chest pain, drowsiness, reduced level of consciousness
18	41–64	Outpatient	Clozapine		None		4.2 (3.6–4.6)	N/A	N/A	Oversedation	Sudden loss of consciousness, confusion, urinary retention
19	41–64	Outpatient	Clonazepam Diazepam Oxazepam	Opioids Topiramate Valproate	C: Valproate–topiramate	Chronic pain post subarachnoid haemorrhage, epilepsy	4.4 (3.6–4.6)	N/A	N/A	Oversedation	Sudden loss of consciousness, falls
20	41–64	Outpatient	Opioids unspecified	Clonazepam Diazepam Oxazepam Valproate Topiramate	C: Valproate–topiramate	Chronic pain post subarachnoid haemorrhage bleeding, epilepsy	3.4 (3.6–4.6)	N/A	N/A	Oversedation	Sudden loss of consciousness
21	41–64	Inpatient	Levomepromazine	Benzodiazepines unspecified	None	COPD, obesity, heart failure	4.3 (3.6–4.6)	N/A	N/A	Oversedation	Sudden loss of consciousness, respiratory depression
22	41–64	Outpatient	Nitrazepam	Alprazolam Clozapine Opioids	C: Alprazolam–clozapine, C: Clozapine–nitrazepam	Kidney cancer, currently post-operative inpatient	3.9 (3.6–4.6)	N/A	N/A	Oversedation	Sudden loss of consciousness
23	≥65	Outpatient	Risperidone	Clomethiazole		Ischaemic heart disease, stroke	4.8 (3.6–4.6)	N/A	N/A	Oversedation	Sudden loss of consciousness, respiratory depression
24	41–64	Outpatient	Clozapine	Zuclopenthixol decanoate LAI im	None	Verified pulmonary embolism days after ADE, unclear whether related	5,6 (3.6–4.6)	N/A	N/A	Oversedation	Loss of consciousness, respiratory depression
25	18–40	Inpatient	Diazepam im	Levomepromazine Zuclopenthixol acetate shorter-acting im	C: Levomepromazine–zuclopenthixol (CYP2D6)	None	4,4 (3.6–4.6)	N/A	N/A	Oversedation	Neurological symptoms, sudden loss of consciousness
26	≥65	Outpatient	Risperidone	Alprazolam Morphine	None	Heart failure, recent infection/pneumonia	4.5 (3.6–4.6)	N/A	N/A	Oversedation	Drowsiness, sudden loss of consciousness
27	18–40	Outpatient	Pregabalin^d^		None	Unclear seizures in the past	4.2 (3.6–4.6)	N/A	N/A	Paradoxical seizure? Unclear	Sudden unconsciousness
28	18–40	Outpatient	Methadone		Unclear	Chronic kidney disease,bowel cancer, intravenous parental nutrition, chronic pain	3.6 (3.6–4.6)	N/A	N/A	QT prolongation	Torsades de pointes QT interval >500 ms
29	≥65	Outpatient	Sertraline		None	Ischaemic heart disease, hypertension	3.9 (3.6–4.6)	N/A	N/A	QT prolongation	Syncope, unclear bradycardia QT interval >500 ms
30	41–64	Inpatient	Levomepromazine		None	History of traumatic brain injury	4.2 (3.6–4.6)	N/A	N/A	Orthostatic hypotension	Drowsiness and instability, fall resulting in a fracture
31	18–40	Inpatient	Quetiapine		None	None	4.1 (3.6–4.6)	N/A	N/A	Cardiac arrhythmia	Tachycardia
32	18–40	Outpatient	Venlafaxine		None		3.8 (3.6–4.6)	N/A	N/A	Malignant hypertension	Systolic BP >200
33	41–64	Outpatient	Clozapine		None		4.1 (3.6–4.6)	N/A	N/A	Suspected orthostatic hypotension	Unclear collapse leading to a fall
34	≥65	Outpatient	Haloperidol po	Clozapine	None	Dementia, lower UTI	4.2 (3.6–4.6)	N/A	N/A	Orthostatic hypotension	Collapse
35	41–64	Inpatient	Tranylcypromine	Sumatriptan	D: Sumatriptan–tranylcypromine	History of traumatic brain injury, migraine	4.4 (3.6–4.6)	N/A	N/A	Hypotension	Hypotonia, drowsiness
36	≥65	Inpatient	Zuclopenthixol po		None	Ischaemic heart disease	4.8 (3.5–4.6)	N/A	N/A	Hypotension	Chest pain, sudden loss of consciousness
37	18–40	Inpatient	Perphenazine po, Perphenazine decanoate LAI im	Alimemazine Clozapine Diazepam Levomepromazine Lithium *Haloperidol short-acting im given 10 days before event*	C: Clozapine–diazepam	None	4.3 (3.5–4.6)	N/A	N/A	Suspected NMS	Drowsiness, falls, sudden loss of consciousness, normal CK values
38	≥65	Inpatient	Zuclopenthixol acetate short-acting im	Haloperidol short-acting im Levomepromazine	C: Levomepromazine–zuclopenthixol (CYP2D6)	Hypertension, hyperlipidaemia, 3^rd^-degree AV block (pacemaker)	5.0 (3.6–4.6)	N/A	N/A	NMS	Loss of consciousness rigidity, rhabdomyolysis, high CK values
39	18–40	Outpatient	Zuclopenthixol decanoate LAI im	Olanzapine po	None		3.7 (3.6–4.6)	N/A	N/A	Acute dystonia in head/neck region	Risk of airway obstruction, rigidity
40	41–64	Outpatient	Haloperidol po		None		4.7 (3.5–4.6)	N/A	N/A	Acute dystonia in head/neck region	Risk of airway obstruction
41	18–40	Outpatient	Sertraline	Tramadol	C: Sertraline–tramadol		3.9 (3.6–4.6)	N/A	N/A	Suspected SS	Confusion, fever, seizures

ADE, adverse drug event; BP, blood pressure; CABG, coronary artery bypass grafting; COPD, chronic obstructive pulmonary disease; C, drug interaction classified as C; D, drug interaction classified as D; DDI, drug–drug interaction; DM2, type 2 diabetes mellitus; K, potassium; im, intramuscular; Li, lithium; NA, non-applicable; NMS, neuroleptic malignant syndrome; NDI, nephrogenic diabetes insipidus; po, per os; PRN, pro re nata; SS, serotonin syndrome; UTI, urinary tract infection; CK, creatinine kinase; intox, intoxication.

a According to case records.

b Janusmed: Online drug/drug interaction (DDI) database published by the health authority of Stockholm Region.

c Lithium therapeutic range 0.4–1.2 mmol/L.

d Classified in ATC substance class N03 during review period. Classified in N02 since 2023.

## 4 Discussion

### 4.1 Findings

In our study, we investigated the incidence of serious ADEs in patients treated for bipolar disease or schizoaffective disorders. Applying the Council for International Organizations for Medicinal Sciences (18) (COIMS) grading, we found that serious ADE overall were uncommon (≥1/1,000 and <1/100) but not rare. Lithium was the single most common cause of a serious ADE and led to a 2.6-times increased risk compared to ADE with no lithium exposure at the time.

One-third of severe ADEs occurred in patients aged 65 years and above, and the IRR for severe ADE in patients 65 years or older was 3.4 times higher. Therefore, severe ADEs should not only be considered in terms of pharmacological effects *per se* but also in relation to decline in liver and/or kidney function occurring with age.

### 4.2 Drug interactions

A combination of several drugs increases the risk of interactions. Such may be pharmacodynamic or pharmacokinetic. A pharmacodynamic interaction occurs when drugs act synergistically, for instance, to cause excessive sedation or respiratory depression. In our study, there were nine such possible interactions, five of which involved opioids given as pain medication. Pharmacokinetic interactions affect serum concentrations. One important cause for such interaction are changes in metabolism, as mediated by the cytochrome P450 (CYP) microsomal enzymes (19). CYP inhibition can increase the concentration of the respective substrates. Examples of clinically relevant CYP inhibitors in the management of bipolar disorder include valproate (CYP2C9), risperidone (CYP2D6), and SSRI such as fluoxetine (CYP2D6). Examples of clinically relevant CYP inductors include carbamazepine and the herbal remedy St. John’s wort (*Hypericum perforatum*) (both CYP3A4). Somatic drugs can also affect plasma concentrations of psychotropic drugs via CYP interactions if these are relevant substrates (20). CYP interactions are partly also genetically determined, which makes them a focus of interest for personalised medicine (21). Therefore, even if the potential for CYP interaction exists theoretically, not all interactions will be of clinical relevance in all patients. Discussing all possible interactions is beyond the scope of this paper, and we refer to relevant reviews in this area (19, 22). In our study, there were 20 events with at least one clinically relevant DDI present according to Janusmed. Only in two of these events was the DDI related to CYP2D6 inhibition. Both concerned a combination of zuclopenthixol with levomepromazine. However, a synergistic neuroleptic effect may also have contributed to these ADEs. In some cases, the significance of the identified DDI was not clear. For instance, for two ADEs, in which opioids and benzodiazepines were judged as primarily implicated, the interaction checker also highlighted a DDI between topiramate and valproate with a risk of encephalopathy. It ultimately remains unclear whether this DDI was of relevance, but it is possible. In many cases, particularly when there is polypharmacy, it can become very difficult to establish with certainty the true mechanism behind a DDI. This becomes even more difficult when there are relevant somatic comorbidities, as for instance present in some of the chronic lithium intoxications.

### 4.3 Comparison with other studies

A few studies have explored emergency or intensive care rates for patients with BD and related disorders (23–26). This may lead to an impression that serious ADEs with psychotropic drugs within the therapeutic drug range are more common than they actually are. The list of adverse drug reactions that could lead to intensive care is long. PRN medication, polypharmacy, antipsychotics, and parenteral formulations are possibly all factors that can increase the risk of serious ADEs and death. However, data remain heterogenous; studies are difficult to compare (27, 28). The relevant literature often takes substances, substance classes, or adverse effects itself as a starting point rather than diagnoses (3, 29, 30).

#### 4.3.1 Lithium and lithium intoxication

Non-intentional lithium intoxications are mostly chronic and can occur in the context of a drug interaction or an adverse effect. Whereas acute intoxications due to overdoses may lead to higher s-lithium, chronic intoxications tend to be more severe since lithium has already passed from the blood to the tissues (31). The proportion between acute and chronic intoxications may vary. One study from a university hospital in Paris found that over a period of 22 years (1992 to 2013), 128 patients had been admitted to intensive care because of intoxication. Of these, 10% were acute-on-lithium-naïve, 63% were acute-on-therapeutic, and 27% were chronic intoxications. This study could not report on the reference population and treatment times (32). This makes it difficult to derive an incidence rate for lithium intoxications requiring intensive care. Using our own LiSIE cohort with a time frame from 1997 to 2017, we found an incidence rate of 9.7/1,000 PY for unintended lithium intoxications (33). In previous work exploring the LiSIE cohort from 1997 to 2013, we found that of 91 identified events of lithium intoxication including overdoses, 34% required intensive care. No fatalities were observed (6). In our study, lithium was causally implicated in 16 of the severe ADEs. Of these, 15 events were due to chronic lithium intoxications. Among these, hypovolaemia may have played a role in several events. The reason for suspected hypovolaemia included infection, fever, hot weather, and lack of oral fluid intake. This highlights the importance of ensuring that individuals treated with lithium are always well hydrated since lithium intoxication is a question of concentration. As kidney function declines with age, elderly individuals may be particularly at risk of chronic lithium intoxication (8). In the presence of somatic comorbidities and/or kidney function decline, a combination of lithium with other drugs that can increase serum lithium concentrations can be particularly hazardous. In our study, one further ADE was due to lithium-induced diabetes insipidus, which persisted after lithium had been discontinued. This then led to hypernatraemia. This event serves as a reminder that lithium-associated adverse events can persist even after lithium has been discontinued (34). Therefore, clinicians should enquire about previous lithium exposure and urinary frequency in individuals with affective disorders.

#### 4.3.2 Torsades de pointes and sudden cardiac death

Many psychotropic medications can prolong the QT interval. This increases the risk of TdP. Substances with a known risk of TdP include some first-generation antipsychotics (FGAs), such as haloperidol; some antidepressants, such as citalopram and escitalopram; and methadone. Substances with a possible risk of TdP include some second-generation antipsychotics (SGA) such as clozapine and lithium. Substances with a conditional risk of TdP include many of the drugs commonly used in the field of psychiatry. Conditional risk means that medicines may cause TdP under special circumstances, i.e., when taken in (a) an excessive dose, (b) conjunction with other drugs that prolong QT, and (c) the presence of additional risk factors, such as congenital long QT interval, extreme bradycardia, or hypokalaemia (35). Prescribing of QT-prolonging medications is common and may increase during inpatient events. A German survey of 27,396 inpatients found that 50% received a combination of at least two QT-prolonging drugs. The odds of receiving such combinations were highest in patients with an F2 diagnosis (schizophrenia, schizotypal, and delusional disorders) and with an F7 diagnosis (intellectual disability) according to the ICD-10 Classification of Mental and Behavioural Disorders (36). In our study, there was one TdP event. This was related to methadone.

#### 4.3.3 Serious adverse drug reactions due to clozapine

Clozapine is associated with a wide range of somatic adverse effects with agranulocytosis and myocarditis being of particular concern (29). It still remains unclear how often clozapine-associated adverse effects become so serious that they warrant intensive or critical care. One retrospective study from the UK found that during a 1-year period in 2018–2019, there were 114 events of hospitalisation for somatic reasons in 87 patients who had been prescribed clozapine. Infections, particularly respiratory, were the most common reason for admission. Three events resulted in intensive care; four events resulted in death. The three intensive care events involved overdose and aspiration pneumonia, respiratory arrest, and status epileptics. The four cases of deaths, none of which had been admitted to critical care, involved lung adenocarcinoma, bowel obstruction, cardiac arrest, and “chest sepsis.” (29) Based on this information, five of the seven (71%) events leading to intensive care or death could have been due to a serious adverse reaction to clozapine. A Canadian study looked at the incidence of myocarditis in clozapine-treated patients who had been admitted to general psychiatric wards between 2016 and 2017. This study found that 10 of 316 patients had myocarditis. Of these, two (20%) patients required critical care. No patient died in the hospital (37). In a French study, 170 clozapine-treated patients were followed over a 11-year period from 1989 to 1999. There were four deaths (2%) definitely attributable to clozapine due to seizures (2), intestinal obstruction (1), and agranulocytosis (1) (38). There was one death possibly attributable to clozapine due to aspiration. In our study, there were three events related to clozapine treatment. All events presented with a loss of consciousness related to a collapse and oversedation. For all cases, we could not establish a clear cardiac aetiology.

#### 4.3.4 Neuroleptic malignant syndrome

NMS is a rare event, but current incidence estimates continue to vary widely, between 0.016 and 0.1% exposed persons (30, 39). One recent study in individuals with schizophrenia or schizoaffective disorder reported incidence rates according to PY exposure. In this study, the incidence rate was 0.199/1,000 PY (40). Mortality figures between 6% and 10% have been reported (5, 41, 42) but may have declined over time (42). In a US nationwide inpatient sample collated over 10 years, the unadjusted NMS-associated mortality declined from 8.3% in 2002 to 5.1% in 2011 (42). In this work, drug overdoses were excluded. Acute respiratory failure, acute kidney injury (AKI), sepsis, and comorbid congestive heart failure were independent predictors of mortality. A recent study has reported an NMS-associated mortality within 30 days of the index event of 6.0% (30). A systematic review of six primary studies and 186 individual cases of NMS suggested that NMS associated with SGA was less common, less severe, and less lethal than NMS associated with FGA (43). A further systematic review analysed 683 cases of NMS for predictors of mortality. This study excluded cases arising from intentional overdoses. Continued antipsychotic treatment, respiratory problems, severity of hyperthermia, and older age were independent predictors of mortality. There was no association with the type of antipsychotic formulation, oral or LAI. Neither was there any association with antipsychotic class, SGA or FGA (44). In our study, there was one event of confirmed and one event of suspected NMS. In both cases, two injectable FGAs had been used close to the event.

#### 4.3.5 Serotonin syndrome

Serotonin syndrome is also a rare event. The incidence of serotonin syndrome has been estimated to be between 0.09% and 0.23% for individuals exposed to serotonergic agents with incidence rates ranging from 1.28/1,000 PY, for exposure to a single serotonergic, non-monoamine oxidase inhibitor agent, to 6.70/1,000 PY, for exposure to ≥ 5 serotonergic, non-monoamine oxidase inhibitor agents (45). The mortality of serotonin syndrome is unknown. One study from the United States (US) indicated a mortality of <1% for serotonin syndrome from overdoses, intentional and unintentional (46). Our own research group conducted a systematic review of serotonin syndrome from 2004 until 2018. Of 412 cases, 173 (42%) were severe having resulted in either intensive care, intubation, coma, or death. Hyperthermia, seen as one hallmark of severe serotonin syndrome, was absent in 27% of 128 severe cases with relevant information available (47). Of the 173 severe cases, 66% were related to surgical procedures, trauma care, overdoses, or substance misuse. Thirty-four percent were related to medical or psychiatric treatment. The five most common reasons of severe serotonin syndrome were non-accidental overdoses, a combination between an antidepressant and methylene blue, a combination of an antidepressant with other psychiatric or somatic drugs, combinations or swaps of antidepressants or dose increase, and a combination of an antidepressant with opioids (47). The event of serotonin syndrome in our study involved a combination of sertraline and tramadol.

#### 4.3.6 Acute dystonia

Acute dystonia involves sustained or intermittent contractions of muscle antagonists leading to slow and twisting movements and abnormal postures (48). Acute dystonia may potentially result from dopaminergic and anticholinergic neurotransmission and is associated with antipsychotics drugs blocking the dopamine-2 (D2) receptor. Acute dystonia may also occur with other drugs, e.g., antiemetics, antidepressants, antihistamines, anticonvulsants, and antimalarials (49, 50). The prevalence of acute dystonia is unknown. One recent study conducted in 441 patients attending a child and adolescent psychiatry department found an incidence of 10.5% for FGA and 2.2% for SGA (51). Further risk factors include young age, male sex, cocaine use, and a history of acute dystonia (50, 51). Acute dystonia can become life threatening when involving laryngo-pharyngeal muscles leading to upper airway obstruction (52). In our study, we identified two such events, both of which involving antipsychotics.

#### 4.3.7 Serious adverse drug reactions due to benzodiazepines

Benzodiazepines are commonly prescribed to individuals with psychiatric disorder. A cross-sectional analysis explored a representative sample of 86,186 US adults from the 2015–2016 National Survey or Drug Use and Health (NSDUH). Nine percent of individuals without any mental illness in the previous year had used benzodiazepines. This compared to 22% benzodiazepine use in individuals with mild mental illness, 29% with moderate mental illness, and 42% with severe mental illness (53). A study of 5,212 adults participating in the US National Health and Nutrition Examination Surveys (NHANES) between 1999 and 2015 explored all-cause mortality in relation to benzodiazepine and opioid use. In this study, 24% of participants had received benzodiazepine prescriptions without concurrent opioids. The death rate was 26.5/1,000 PY. This compared to a death rate of 20.2/1,000 PY in participants treated with selective serotonin reuptake inhibitors. The study did not distinguish between overdoses and adverse drug reactions or drug interactions (54). In our study, there were seven events of oversedation related or possibly related to benzodiazepines.

#### 4.3.8 Serious adverse drug reactions due to opioids

In our study, there were seven severe ADEs linked to opioids. Opioids are not used in the treatment of BD and SZD itself. However, medical opioid use has been shown to be common among individuals with psychiatric disorders, for instance, in the context of chronic pain (55). Therefore, we include opioids in our discussion here. One study from the US, using data from the Medical Expenditure Panel Survey (MEPS) for the years 2011 and 2013, estimated by extrapolation that among 39 million Americans with mental health disorders, 19% used prescription opioids. Prescription opioid use was commonest in individuals with depression and anxiety. Opioids were mostly prescribed for musculoskeletal conditions (55). A further study based on MEPS data explored prescription opioid use among construction workers, using a similar method. This study found that 10% had used prescription opioids. Prescription opioid use was higher in individuals having experienced a work-related injury, non-work-related injuries, musculoskeletal disorders, and poorer physical or mental health disorders (56). Opioids may interact with a range of other psychotropic medicines. For instance, co-administration with benzodiazepines may lead to central nervous system (CNS) and respiratory depression. A retrospective population-based study from Canada compared clinical outcomes in individuals using opioids only and individuals using opioids and benzodiazepines concurrently. Risk of death increased by 90% in individuals using benzodiazepines and opioids concurrently compared to individuals with opioid-only prescriptions (57). In the previously mentioned study based on NHANES, 38% used opioids only, and 9% used opioids in combination with benzodiazepines. The death rates were 22.8/1,000 PY in individuals with opioids only and 33.0/1,000 PY in individuals with concurrent treatment with opioids and benzodiazepines (54). Opioids that inhibit serotonin reuptake, such as tramadol, pethidine (meperidine), pentazocine, and dextromethorphan, can interact with antidepressants. In our own review of 173 cases with severe serotonin syndrome, 9.2% had been due to a combination between opioids and antidepressants (47).

### 4.4 Strengths

With access to prescriptions, laboratory data, and medical records, we were able to validate diagnoses, establish the chronology of events, and calculate incidence rates in PY based on mood-stabilising treatment. The validation of laboratory and/or prescription data increased our ability to judge whether serious ADE was truly related to psychotropic treatment, thereby reducing the potential for misclassification and overestimation beyond what is possible in observational studies based on register data.

### 4.5 Weaknesses

The nature of our study was observational and retrospective. Relying on medical records meant that the quality of our study depended on the quality of the information recorded. However, serious ADEs are notable events that tend to be carefully recorded, are uncommon, and can occur at any time. Therefore, they are difficult to study in a prospective study or a randomised controlled trial. For schizoaffective disorder, we did not distinguish between affective subtypes, relying on earlier diagnosis validation in the LiSIE study. However, we judge that making such a distinction would not have affected the results.

## 5 Conclusion

Serious ADEs related to treatment of BD or SZD were uncommon but not rare. Older individuals were particularly at risk. The risk was higher in individuals exposed to lithium. Serum lithium concentration should always be checked when patients present with new or unclear somatic symptoms. However, severe ADEs also occurred with other mood stabilisers and other psychotropic drugs. For these, oversedation and cardiac and blood pressure-related events were commonest.

## Data availability statement

The datasets generated and/or analysed during the current study are not publicly available due to lack of ethics committee permission and not having been part of the consent process. The structure of the dataset and the coding specification are available from the authors. Any other reasonable request will be raised with the healthcare provider. Requests to access the datasets should be directed to petra.truedson@umu.se.

## Ethics statement

The studies involving humans were approved by the Regional Ethics Review Board at Umeå University, Sweden. The studies were conducted in accordance with the local legislation and institutional requirements. The ethics committee/institutional review board waived the requirement of written informed consent for participation from the participants or the participants' legal guardians/next of kin because all participants were informed about the nature of the study in writing and provided verbal informed consent. The consent was documented in our research files, dated, and signed by the research worker who obtained the consent. In accordance with the ethics approval granted, deceased patients were also included. This procedure was approved by the Regional Ethics Review Board.

## Author contributions

PT: Conceptualization, Formal analysis, Methodology, Validation, Writing – original draft, Writing – review & editing. MO: Conceptualization, Data curation, Formal analysis, Methodology, Supervision, Validation, Writing – review & editing. LW: Methodology, Validation, Writing – review & editing. RL: Methodology, Writing – review & editing. MM: Methodology, Writing – review & editing. KL: Methodology, Writing – review & editing. IL: Methodology, Validation, Writing – review & editing. UW: Conceptualization, Data curation, Formal analysis, Methodology, Supervision, Validation, Writing – original draft, Writing – review & editing.

## Supplementary material

Appendix 1 STROBE checklist.
