# Supplementary material for: Serious adverse drug events associated with psychotropic treatment of bipolar or schizoaffective disorder: a 17-year follow-up on the LiSIE retrospective cohort study
Source: Front Psychiatry. 2024 Apr 3;15:1358461. doi: 10.3389/fpsyt.2024.1358461 (PMC11022285; doi:10.3389/fpsyt.2024.1358461)
Supplement: Appendix 1 — STROBE checklist. [file DataSheet_1.pdf]

## Appendix 1: STROBE Statement - checklist for our study

| **STROBE requirement** | **#** | **Our study** |
|---|---|---|
| *Title and abstract* | 1 | |
| *(a)* Indicate the study's design with a commonly used term in the title and abstract | | (a) Given: *Serious adverse drug events associated with psychotropic treatment of bipolar or schizoaffective disorder: A 17-year follow-up on the LiSIE retrospective cohort study* |
| *(b)* Provide in the abstract an informative and balanced summary of what was done and what was found | | (b) Structured abstract provided. |
| *Introduction* | | |
| Background/rationale: Explain the scientific background and rationale for the investigations being reported | 2 | Background outlined in introduction. |
| Objectives: State specific objectives, including any pre-specified hypotheses | 3 | Aims clearly stated in text, "The aims of this study were for patients with BD or SZD to (a) determine the incidence of serious ADE, (b) compare the incidence rates of serious ADE caused by lithium with ADE caused by other psychotropic medications, and (c) describe the aetiology of the serious ADE identified". |
| *Methods* | | |
| Study design: Present key elements of the study design early in the paper | 4 | Study design: Retrospective cohort study.<br>Key elements of the study included in the manuscript: study design, ethics and consent, sample, patient selection and inclusion criteria, exclusion criteria, outcome definition, exposure parameters, time in study, validation process, medical chart review, control for bias, missing data and statistical analysis. |
| Setting: Describe the setting, locations, and relevant dates, including periods of recruitment, exposure, follow-up, and data collection | 5 | Setting and all relevant dates described in manuscript:<br>"LiSIE invited all adults in the regions Västerbotten and Norrbotten who had either received a diagnosis of BD (ICD10 F31) or schizoaffective disorder (SZD) (ICD10 F25) according to the 10th revision of the International Statistical Classification of Diseases and Related Health Problems or who had used lithium as a mood-stabiliser between 1997 and 2011.<br><br>The current study included patients from the Norrbotten region, having received a diagnosis of either BD or SZD on at least two occasions at least 180 days apart. In line with the ICD-10 classification, we also included patients under the BD category, when they had been diagnosed with at least one manic and one depressive event. For this study, we screened all events of critical, post-anaesthesia, or intensive care documented in the medical records. We only included events related to an unintended serious ADE involving a psychotropic drug in its own right or in terms of a drug interaction. The outcome serious ADE was determined over a 17-year period from 1st January 2001 to 31st December 2017.<br><br>For the whole LiSIE study, we excluded patients in whom, after manual medical record validation, a diagnosis of schizophrenia or personality disorder was more likely than BD or SZD. For the current study, we excluded patients in whom ADE occurred during care under |

| | | |
|---|---|---|
| | | forensic services; we had not applied for ethical approval to access these records.” |
| Participants:<br>*(a)* Give the eligibility criteria, and the sources and methods of case ascertainment and control selection. Give the rationale for the choice of cases and controls<br>(*b*) For matched studies, give matching criteria and the number of controls per case | 6 | (a) As above, cf. 5. The medical records of all eligible patients were retrospectively reviewed for the outcomes and variables under study, from 1 January 2001 up to 31 December 2017**.**<br><br>(b) N/A. |
| Variables:<br>Clearly define all outcomes, exposures, predictors, potential confounders, and effect modifiers. Give diagnostic criteria, if applicable | 7 | Described in manuscript. “The main exposure parameter was psychotropic drugs. To characterise the serious ADE, we checked for use of PRN, polypharmacy, and use of parenteral psychotropic drugs, the latter either in form of long-acting injectable antipsychotics (LAI) or acute injections. Polypharmacy was defined as concurrent use of $\geq$3 psychotropic medicines. For inpatients, we were not able to establish doses because these fluctuated and documentation was not complete. For outpatients, we recorded doses given on the prescription at the time of the ADE. We also explored use of somatic drugs that could have interacted and somatic comorbidities that could have been related to the ADE.<br><br>Other exposure parameters included age, sex, and type of underlying mood disorder. For subcategories of mood disorder, we relied on previous validation of the LiSIE cohort that had explored how diagnoses would have looked according to DSM-5, BD (296.4, 296.80, 296.89) or SZD (295.7). This validation had used medical records until 31 December 2015. We also checked for serum lithium concentrations and serum creatinine concentrations at the time of the serious ADE. We also checked serum potassium concentrations as a risk factor for arrythmias. Finally, we divided patients in four groups according to the role lithium exposure might have played; (A) lithium exposure at the time and causally implicated, (B) lithium exposure at the time but not causally implicated, (C) no lithium exposure at the time, and (D) no lithium exposure at the time but previous lithium causally implicated. |
| Data sources /measurement:<br>For each variable of interest, give sources of data and details of methods of assessment (measurement). Describe comparability of assessment methods if there is more than one group | 8 | Definition for each variable given in text.<br>“For the outcomes and exposure variables, we retrospectively reviewed the medical records of all eligible patients from 1st January 2001 to 31st December 2017. From the medical records, we manually validated serious ADE and the concurrent lithium treatment. At that point, based on the information in the medical records, we determined the most likely cause of the ADE. In patients treated with lithium at the time, we evaluated whether lithium was (a) causally implicated in the ADE, (b) used at the time but not implicated in the ADE, or (c) not used. For all ADE, we also recorded somatic comorbidities present at the time, which could have possibly contributed to the event. Two authors with psychiatric background (PT and UW) conducted the validation of ADE events. When uncertain, |

| | | |
|---|---|---|
| | | they consulted with a third author with medical background (MO). To ensure that we did not miss any potential drug interactions, including cytochrome 450 (CYP) mediated interactions, we used the "Janusmed" interaction checker." |
| Bias:<br>Describe any efforts to address potential sources of bias | 9 | Described in manuscript.<br>Potential sources of bias discussed, including selection and observer bias. "We had controlled for selection bias in the whole LiSIE study with key parameters available in anonymised form. These included age and sex. Where applicable we also controlled for maximum recorded concentrations of lithium and creatinine. In accordance with the ethics approval granted, we had compared these parameters for consenting and non-consenting patients. No significant differences were found between the two groups. The data was complete for included patients for the defined outcome. For some patients, not all medications could be derived from the prescription module. For these, we could not accurately establish the dose. For some drugs we could not establish with certainty involvement in the severe ADE. Such we reported under "other potentially contributing medications" in the table listing the ADE." |
| Study Size:<br>Explain how the study size was arrived at | 10 | Cf. figure 1 "Selection of study sample" |
| Quantitative variables:<br>Explain how quantitative variables were handled in the analyses. If applicable, describe which groupings were chosen and why | 11 | Exposure variables described in detail in the text. Handling of variables described in statistical methods as below.<br><br>"The data were anonymised before analysis. Then, the data were analysed descriptively. We calculated the incidences for serious ADE with and without lithium being causally implicated and for age <65 or ≥65 years. We then calculated the incidence rate ratio (IRR) and 95% confidence intervals (CI)."<br><br>We also described serious ADE regarding relevant exposure parameters stratified by lithium exposure. As the sample size was small, we used non-parametric methods. We used Fisher's Exact test to compare sex and treatment setting and Mann-Whitney U test to compare age. We did not conduct any further multivariate analysis to avoid overfitting, i.e. fitting too many variables in relation to the small sample size." |
| Statistical methods:<br>*a)* Describe all statistical methods, including those used to control for confounding<br>*(b)* Describe any methods used to examine subgroups and interactions<br>(*c)* Explain how missing data were addressed<br>*(d)* If applicable, explain how matching of cases and controls was addressed | 12 | (a) The data were reported at episode level. The data were anonymised before analysis. Then, the data were analysed descriptively. We calculated the incidences for serious ADE with and without lithium being causally implicated and for age <65 or ≥65 years. We then calculated the incidence rate ratio (IRR) and 95% confidence intervals (CI). We also described serious ADE regarding relevant exposure parameters stratified by lithium exposure. As the sample size was small, we used non-parametric methods. We used Fisher's Exact test to compare sex and treatment setting and Mann-Whitney U test to compare age. We did not conduct any further multivariate analysis to avoid overfitting, i.e. fitting too many variables in relation to the small sample size. |

| | | |
|---|---|---|
| *(e)* Describe any sensitivity analyses | | The data was processed with SPSS version 27.0 (IBM, Armonk, NY, USA) and MedCalc Software Ltd (Version 20.116). The significance level was set at a p-value of 0.05 throughout.<br><br>(b) Described in text. “We also described serious ADE regarding relevant exposure parameters stratified by lithium exposure. As the sample size was small, we used non-parametric methods. We used Fisher’s Exact test to compare sex and treatment setting and Mann-Whitney U test to compare age. We did not conduct any further multivariate analysis to avoid overfitting, i.e. fitting too many variables in relation to the small sample size.”<br><br>(c) Addressed in the text. “The data was complete for included patients for the defined outcome. For some patients, not all medications could be derived from the prescription module. For these, we could not accurately establish the dose. For some drugs we could not establish with certainty involvement in the severe ADE. Such we reported under “other potentially contributing medications” in the table listing the ADE”.<br>(d) N/A<br>(e) N/A |
| *Results* | | |
| Participants:<br>*(a)* Report numbers of individuals at each stage of study—eg numbers potentially eligible, examined for eligibility, confirmed eligible, included in the study, completing follow-up, and analyzed<br>*(b)* Give reasons for non-participation at each stage<br>*(c)* Consider use of a flow diagram | 13 | (a+b) “1,521 patients were included. Of these, 945 (62.1%) were female and 576 (37.9%) were male; 1,298 (85.3%) had a diagnosis of BD and 223 (14.7%) a diagnosis of schizoaffective disorder. During the whole review period, 841 (55.3%) patients had been exposed to lithium at any time. In terms of observation time, there were 21,977 PY available. 5,586 PY with lithium exposure, 16,391 PY without lithium exposure, 19,038 PY for age <65 years and 2,939 PY for age ≥65 years.<br><br>In total, there were 37 patients, 14 men and 23 women, who had experienced 41 events of serious ADE”<br><br>(c) Flow chart included in the manuscript as figure 1. |
| Descriptive data:<br>*(a)* Give characteristics of study participants (e.g. demographic, clinical, social) and information on exposures and potential confounders<br>*(b)* Indicate number of participants with missing data for each variable of interest | 14 | (a) Baseline characteristics described in table 2 of the manuscript.<br><br>(b) Included in the flow chart (figure 1) and in the text. |
| Outcome data:<br>Report numbers in each exposure category, or summary measures of exposure | 15 | Outcome data presented in text and table 2,3,4 |

| | | |
|---|---|---|
| Main results<br>*(a)* Give unadjusted estimates and, if applicable, confounder-adjusted estimates and their precision (eg, 95% confidence interval). Make clear which confounders were adjusted for and why they were included<br>*(b)* Report category boundaries when continuous variables were categorized<br>*(c)* If relevant, consider translating estimates of relative risk into absolute risk for a meaningful time period | 16 | (a) Results presented according to the statistical method outlined in item 12<br><br>(b) Results presented according to the statistical method outlined in item 12. Variable definitions given in method.<br><br>(c) N/A |
| Other analysis:<br>Report other analyses done—e.g. analyses of subgroups and interactions, and sensitivity analyses | 17 | Subgroup-analysis of serious ADE regarding relevant exposure parameters stratified by lithium exposure. Cf. 12. |
| *Discussion* | | |
| Key results:<br>Summarize key results with reference to study objectives | 18 | Done |
| Limitations:<br>Discuss limitations of the study, taking into account sources of potential bias or imprecision. Discuss both direction and magnitude of any potential bias | 19 | Discussed in text. "The nature of our study was observational and retrospective. Relying on medical records meant that the quality of our study depended on the quality of the information recorded. However, serious ADE are notable events that tend to be carefully recorded, are uncommon and can occur at any time. Therefore, they are difficult to study in a prospective study or a randomised controlled trial. For schizoaffective disorder, we did not distinguish between affective subtypes, relying on earlier diagnosis validation in the LiSIE study. However, we judge that making such a distinction would not have affected the results." |
| Interpretation:<br>Give a cautious overall interpretation of results considering objectives, limitations, multiplicity of analyses, results from similar studies, and other relevant evidence | 20 | Results discussed in view of the limitations (weaknesses) of our study design and use of medical case records. |
| Generalisability:<br>Discuss the generalizability (external validity) of the study results | 21 | Discussed in the context of bias, cf item 9. |

| | | |
|---|---|---|
| Funding:<br>Give the source of funding and the role of the funders for the present study and, if applicable, for the original study on which the present article is based | 22 | Research & Development Fund of Norrbotten, Research and Innovation Unit NLL-941888, NLL-969413 and NLL-969485; The Department of Psychiatry, Sunderby Hospital, Region of Norrbotten. Joint research fund of the Swedish state and the Swedish Health Authorities (ALF), Västerbotten County, RV-939217. Umeå University Insamlingsstiftelse research fund 310157001.<br><br>Conflict to interest statement for all authors included in manuscript. |

Source: http://www.strobe-statement.org/. Accessed 18 December 2023
